# Electrical Stimulation-Guided Approach to Pulmonary Artery Catheter Ablation in Patients with Idiopathic Pulmonary Arterial Hypertension: A Pilot Feasibility Study with a 12-Month Follow-Up

**DOI:** 10.1155/2020/8919515

**Published:** 2020-02-17

**Authors:** Natalia S. Goncharova, Olga M. Moiseeva, Heber Ivan Condori Leandro, Irina S. Zlobina, Aelita V. Berezina, Kirill N. Malikov, Dmitry M. Tashkhanov, Dmitry S. Lebedev, Evgeny N. Mikhaylov

**Affiliations:** ^1^Noncoronary Heart Disease Department, Almazov National Medical Research Centre, Saint Petersburg, Russia; ^2^Neuromodulation Laboratory, Almazov National Medical Research Centre, Saint Petersburg, Russia; ^3^Circulation Physiology Department, Almazov National Medical Research Centre, Saint Petersburg, Russia; ^4^Anesthesiology and Intensive Care Department, Almazov National Medical Research Centre, Saint Petersburg, Russia; ^5^Arrhythmia Department, Almazov National Medical Research Centre, Saint Petersburg, Russia; ^6^Department of Bioengineering Systems, Saint-Petersburg Electrotechnical University “LETI”, Saint Petersburg, Russia

## Abstract

**Background:**

Recently, transcatheter pulmonary artery (PA) ablation aiming at sympathetic denervation has been proposed in pulmonary arterial hypertension (PAH). This pilot feasibility study aimed to assess the feasibility of selective radiofrequency PA ablation based on response to high-frequency stimulation mapping.

**Methods:**

The study comprised 3 female patients with idiopathic PAH (IPAH). The following reactions to PA stimulation were noted and marked by color points on the three-dimensional map: sinus bradycardia (heart rate decrease ≥15%), tachycardia (heart rate increase ≥15%), phrenic nerve capture, and cough. Since the most appropriate ablation strategy was unknown, two approaches were suggested, according to stimulation results: ablation at points with any heart rate response (either bradycardia or tachycardia)—this approach was applied in patient #1 (IPAH long-term responder to calcium channel blockers); segmental ablation at points with no response and with tachycardia response (one IPAH long-term responder to calcium channel blockers patient and one–IPAH with negative vasoreactive testing). Hemodynamic measurements were performed before and after denervation. Follow-up visits were scheduled at 6 and 12 months.

**Results:**

Six-months follow-up was uneventful for patients #1 and 3; patient #2 had one syncope and reduced 6-minute walk test distance and peak VO_2_ consumption. At 12 months, there was a normalization of mean PA pressure and pulmonary vascular resistance (PVR) in patient #1. Patient #2 had no change in PA pressure and PVR at 12 months. Patient #3 remained in II functional class; however, there was an increase in mean PA pressure and loss of vasoreactivity.

**Conclusions:**

Electrical high-frequency stimulation of the PA identifies several types of evoked reactions: heart rate slowing, acceleration, phrenic nerve capture, and cough. The improvement in clinical and hemodynamic parameters following targeted PA ablation in the IPAH patient with positive vasoreactive testing should be confirmed in larger studies.

## 1. Introduction

Pulmonary arterial hypertension (PAH) is a pathophysiological condition with an unfavorable outcome. Current PAH therapy targets three main pathways connected with endothelial dysfunction responsible for pulmonary artery (PA) remodeling. This includes inhibition of vasoconstrictor substances and activation of the main vasodilators, such as nitric oxide and prostacyclin. Despite the widespread introduction of PAH specific therapy, patient mortality remains high accounting for about 10% patient-year [1, 2], and 5-year survival is still unacceptably low (59.4%) [3]. Along with the autocrine and paracrine factors, the autonomic nervous system plays a major role in the regulation of pulmonary circulation [4]. Pulmonary vasculature is innervated by vagus nerve branches, stellate ganglion branches, and by cervicothoracic sympathetic fibers, which include adrenergic, cholinergic, and sensory fibers. The perivascular neural net is formed by fibers from the hilum of the lung and is located in the PA adventitia; the maximum nerve concentration is decreased from proximal to distal PA. The majority of PA nerves are sympathetic fibers [5, 6].

Recently, transcatheter PA radiofrequency ablation at its bifurcation aiming sympathetic denervation has been proposed in experimental PAH [7]. In these studies, PA pressure decrease has been documented following circular ablation of the PA bifurcation and proximal parts of the left and right main PAs [8, 9]. Clinical studies have been published demonstrating significant PA pressure decrease and clinical improvement following nonselective circular PA ablation [10].

However, extended circular PA ablation is performed regardless of the preferential distribution of nerves in PA adventitia, and might be associated with further PA fibrotic remodeling, and loss of PA distensibility. There is a lack of information on how to perform targeted and sufficient PA denervation.

Theoretically, electrical high-frequency stimulation of nerve fibers and ganglia, performed from the PA endothelial surface, might be useful for sympathetic and parasympathetic neural elements differentiation. Beyond proprietary PA neural elements, this stimulation may reveal other major nerves in the mediastinum that can be located near enlarged PA in PAH patients, such as the phrenic and left laryngeal recurrent nerves. We suggest that more selective ablation at sites with autonomic evoked responses to high rate stimulation might be safer than the “anatomic” approach with extended circular PA ablation.

This pilot study was to assess the feasibility of segmental radiofrequency PA ablation based on response to high-frequency stimulation mapping.

## 2. Methods

### 2.1. Patient Characteristics

This pilot feasibility study comprised 3 female patients with idiopathic PAH (IPAH). The study protocol and patient informed consent were reviewed and approved by the Almazov National Research Centre ethics committee.

Inclusion criteria were the following: patients with IPAH in stable condition and constant PAH-specific therapy within at least 3 months before inclusion. IPAH diagnosis was established according to the current clinical practice guidelines [2]. The patients provided signed informed consent to participate in the study. Exclusion criteria were IV functional class (FC) PAH, unstable hemodynamics, absence of PAH specific therapy, sustained atrial tachyarrhythmia (atrial flutter/fibrillation, atrial tachycardia), acute inflammatory diseases, unstable concomitant diseases, psychological disorders, and pregnancy/breastfeeding. Patients #1 and #3 were classified as being long-term responders to calcium channel blockers (CCB) therapy [11]. These both patients were in II FC IPAH according to the World Health Organization (WHO) and received CCB: amlodipine and diltiazem, respectively. The IPAH patient #2 was in III FC (WHO) and received a combination of sildenafil and ambrisentan. None of the patients received antithrombotic therapy.

Patient demographic and clinical characteristics are presented in Table 1.

### 2.2. Pulmonary Artery Stimulation Mapping and Ablation

**Figure 1:**
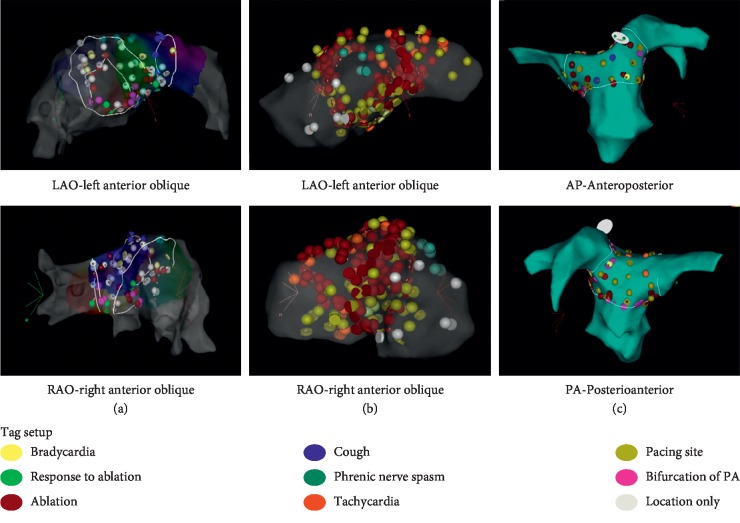
Three-dimensional reconstruction of the PA trunk and PA bifurcation with point tags representing reactions to stimulation. A, B, C panels represent patients 1, 2, and 3, correspondingly.

The mapping and ablation procedure was performed in an electrophysiology laboratory in a fasting state. The right femoral access was performed under local anesthesia. A multipurpose 8 F nonsteerable sheath was introduced into the PA (Preface Multipurpose, Cordis, USA) and then an irrigated 3.5 mm tip ablation catheter (NaviStar ThermoCool, Biosense Webster, USA) with a magnetic sensor was introduced. The procedure was performed under electroanatomical guidance (CARTO 3, Biosense Webster, USA) with three-dimensional reconstruction of the PA trunk, its bifurcation, and proximal parts of the right and left PAs. Stimulation was performed within the area 15 mm proximal to the bifurcation and 15 mm distal the bifurcation, with 5-6 mm distance between each stimulation point. Each stimulation point was marked at the electroanatomical map. Stimulation was performed using the ablation catheter connected to the programmable electrophysiological stimulator EPS320B (Micropace, Australia). The electrical current was set at 33 Hz, 15 mA, and pulse width 1 ms; trains of stimulation were 7–10 s long. Before each high-frequency stimulation train, pacing at 500 cycle length was applied first in order to exclude eventual atrial or ventricle myocardium capture and to prevent tachyarrhythmia induction by direct myocardial stimulation. The following reactions to stimulation were noted and marked by color points on the map: sinus bradycardia (abrupt decrease in heart rate ≥15%), tachycardia (abrupt increase in heart rate ≥15%), phrenic nerve capture, and cough (Figure 1).

Since the most appropriate ablation strategy was unknown at the beginning of the study, two approaches were suggested, according to stimulation results. A more limited approach with ablation at points with either bradycardia or tachycardia response–this approach was applied in patient #1. A more extended approach with segmental ablation at points with both no response and tachycardia response to stimulation; this approach was suggested to result in higher sympathetic denervation and to avoid parasympathetic denervation. In all patients, ablation was avoided at sites with phrenic nerve capture and cough during stimulation. Radiofrequency ablation was performed using the Stockert radiofrequency generator (Biosense Webster, USA) with the following parameters: 30 W, irrigation 17 ml/min, applications 15–30 s. After stimulation and before the first ablation intravenous fentanyl was administered (0.1 mg bolus with additional administrations when required).

After PA ablation vascular sheaths were withdrawn; all patients were observed in an intensive care until the next morning. Anticoagulation with a nonvitamin K oral anticoagulant was initiated after hemostasis was achieved in the groin and was continued 2 months to prevent potential PA thrombosis at ablation sites. The patients were discharged after 3 days.

### 2.3. Hemodynamic Evaluation

Hemodynamic measurements were performed via the right subclavian approach using the 7F Swan Ganz catheter (Corodyn TD, B. Braun Medical Ltd., Germany). An ArterioFix catheter (B. Braun Medical Ltd., Germany) was placed into the left radial artery. Blood samples for oxygen saturation assessment were taken from the pulmonary artery and radial artery. Invasive hemodynamics and oximetry were measured before PA ablation, immediately after ablation, at 6 months (in one patient) and at 12 months (in all 3 patients). Cardiac output (CO) was determined using the Fick equation. Pulmonary vascular resistance (PVR) was calculated using the following formula: PVR = [80 × (mean PA pressure − PCWP)/CO], where CO–cardiac output, PCWP–pulmonary capillary wedge pressure. Acute vasoreactivity testing with 5 mcg iloprost inhalation was repeated in patients #1 and 3, who received CCB therapy.

### 2.4. Follow-Up

Follow-up visits were scheduled at 6 months postablation, and planned hospitalization with cardiac catheterization was performed at 12 months. At visits, standard clinical evaluation, NT-proBNP test (Elecsys, Roche Diagnostic GmbH, Germany), transthoracic echocardiography (VIVID 7 system, GE, USA), six-minute walk test, and cardiorespiratory testing (Oxycon Pro, CARDINAL HEALTH, Germany) were performed.

## 3. Results

Results of PA stimulation and radiofrequency ablation data are presented in Table 2. Hemodynamic parameters measured at baseline, immediately after ablation, at 6 months and 12 months of follow-up, are presented in Table 3.

During PA ablation all patients experienced chest pain requiring intravenous fentanyl administration.

### 3.1. Acute Hemodynamic Changes after Ablation

Following PA ablation acute hemodynamic changes were different among patients (Table 2). However, there were no clinically significant changes in the pulmonary or systemic circulation.

### 3.2. Six-Months Follow-Up

There were no hospitalizations during the first 6 months following ablation. Patient #1: There was an improvement in six-minute walk test distance, NT-proBNP level was decreased, and right atrial area and right ventricle sizes were reduced (Table 3). Patient #2: Reported one syncope on physical exertion at 1 month after ablation. Unscheduled lung *V*/*Q* scan ruled out pulmonary thromboembolism. During the six-minute walk test, there was a significant reduction in walking distance, an increase in NT-proBNP level, and an increase in the right-left ventricle diameter ratio (Table 3). Patient #3: There was an increase in peak VO_2_ consumption during cardiorespiratory testing, a decrease in NT-proBNP level, and a decrease in the right-left ventricle ratio (Table 3). However, the patient reported mild worsening of dyspnea; *V*/*Q* scan ruled out pulmonary embolism, and pulmonary function test showed no changes. Catheterization with the vasoreactive testing was repeated and showed a positive response; therefore CCB therapy was continued.

### 3.3. Twelve-Months Follow-Up

Patient #1 remained physically active with high exercise tolerance (Table 3); echocardiography showed reverse remodeling of right heart chambers and improvement in right ventricle contractility. There was a further decrease in NT-proBNP level (Table 3). Repeat catheterization showed normalization of mean PA pressure and PVR (Table 2).

Patient #2 showed further PAH worsening with six-minute walk distance decrease, ascites, hepatomegaly, right atrial and right ventricle enlargement with right ventricle function decline and pericardial effusion, worsening of cardiac output, and hypoxemia. Accordingly, no change in PA pressure and PVR was noted.

Patient #3 remained in II FC (WHO) without signs of right heart failure or syncope. However, the patient complained decline in exercise tolerance, confirmed on the six-minute walk test and cardiorespiratory testing (Table 3). Echocardiography showed right atrial enlargement and reduction of the right ventricle systolic function (Table 3). Catheterization showed an increase in mean PA pressure and negative vasoreactive test. Therefore, diltiazem was withdrawn and PAH specific therapy was initiated (sildenafil) with further significant improvement in exercise tolerance.

### 3.4. Adverse Events

A groin hematoma was noted in patient #2. Hemorrhagic events were encountered in two patients within 2 weeks after discharge while on oral anticoagulation with apixaban 5 mg BID: patient #1 noted unusually extensive menorrhea; patient #2 experienced nasal bleeding. No additional intervention was required in any patient. At 2 months after ablation oral anticoagulation was ceased and no further bleeding was noted.

## 4. Discussion

The main finding of our pilot study is that stimulation mapping of neural elements of the PA itself and the main nerves around the PA is feasible and provides novel insights into anatomical correlations. The stimulation of PA has evoked several important reactions. Phrenic nerve capture indicates the close proximity of the left phrenic nerve to the PA. Therefore, we suggest that the “anatomical” approach to circumferential PA ablation might lead to inadvertent phrenic nerve damage. Application of stimulation mapping and tagging of phrenic nerve course on the PA map might be helpful in delineating sites to avoid harmful ablation. The same principle can be applied to sites of stimulation-evoked cough. Cough may reflect activation of the laryngeal recurrent nerve, adjacent to the left PA, or, less likely, direct irritation of bronchial nerve branches. Avoidance of ablation at these sites may preserve unnecessary collateral nerve damage.

Heart rate changes during stimulation were prominent and had two different types: rate slowing and rate acceleration. In our opinion, these reactions cannot be attributed to parasympathetic or sympathetic activation solely. Considering the relatively dense distribution of nerves in the PA bifurcation adventitia [9, 12], concomitant activation of parasympathetic ganglia and sympathetic nerve sproutings by stimulation from a relatively large 3.5 mm tip catheter is expected. Therefore, we suggest that any change in heart rate during stimulation might reflect unselective autonomic nerve irritation. On this basis, ablation performed in patient #1 might be more appropriate than more extended ablation performed in patients #2 and #3, where only sites with phrenic nerve capture and cough were avoided. The extended ablation approach should lead to a higher level of denervation, as described in left atrial ganglia ablation studies [13, 14]. On the other hand, the extended “anatomical” approach may be associated with injury of PA wall and lead to its remodeling and further loss of PA distensibility, which is associated with worse prognosis in PAH [15, 16].

It should be acknowledged that there were no systemic blood pressure reactions and no change in PA pressure during stimulation mapping. This is not in line with a previous report of PA stimulation in a patient with IPAH, where stimulation was associated with pressure changes [17].

Acute mild PA and systemic pressure changes immediately after ablation might be associated with fluid infusion and pain reaction during radiofrequency energy delivery.

We speculate that significant clinical improvement in patient #1 is directly related to the ablation procedure. The patient had a long history of IPAH (13 years) with a slow but progressive worsening of symptoms in due course. Since disease progression in these patients is inevitable [2], clinical improvement after ablation on the same medical therapy should be judged as related to the invasive procedure. Importantly, patient #1 had a long-term response to CCB, which is thought to be genetically determined and make PAH progression different from other patients [18]. This difference is related to genes encoding vascular smooth muscle cell contraction and cell calcium metabolism [19]. Single reports showed isolated hypertrophy of the smooth muscle layer of the PA with minimal involvement of intima in vasoresponders, while in patients with a negative vasoreactive test, thickening and fibrosis of endothelial layer are described [20, 21]. In this regard, a decrease in the degree of the smooth muscle layer hypertrophy and suppression of vasospasm by reducing the sympathetic nervous activity may be a justification for the PA denervation method in vasoresponders. However, the contribution of the autonomic nervous system to the regulation of vascular tone in PAH vasoresponders has not been studied. There is no previous clinical experience in PA denervation in PAH patients with positive vasoreactivity testing.

On the other hand, the reason for the disappearance of vasoreactivity and deterioration of clinical and hemodynamic state in patient #3 remains unclear. This might be associated with a too large ablated PA area, or with the natural progression of IPAH. Among IPAH patients with a positive vasoreactive testing, only 6.8% of patients remain long-term responders to CCBs [22].

Various PA denervation approaches are based on animal experiments and cannot be fully extrapolated to patients with PAH. Information on the distribution of sympathetic and parasympathetic nerve fibers and ganglia in the human PA is scarce. Circular ablation of the PA trunk and its main branches with an intention to reduce PA pressure is a more empirical approach. Nevertheless, despite the variability in the number and location of the ablation points proposed by different authors, as well as variable radiofrequency ablation power and duration of each application, and even energy source (radiofrequency, ultrasound), researchers report on positive effects of PA denervation in a significant number of patients with different types of pulmonary hypertension [10, 23–27].

## 5. Study Limitations

The major limitation of this pilot feasibility study is the limited number of patients. However, this is an explorative study and given potentially unknown effects of stimulation-guided PA ablation, a larger study population at this stage might have been unjustified. Our main aim was to assess the feasibility and potential applicability of electrical stimulation-guided PA ablation in IPAH patients. The results of PA stimulation mapping in patients with other PAH types could be different due to the different neural regulations in the pathogenesis of the disease.

Another limitation is that we are unable to distinguish between the natural PAH progression and the adverse effects of PA denervation in two patients since this was a pilot observational study without a control group.

## 6. Conclusions

According to the results of this pilot feasibility study, electrical high-frequency stimulation of the PA bifurcation and proximal PA branches identifies several types of evoked reactions: heart rate slowing, acceleration, phrenic nerve capture, and cough. The improvement in clinical and hemodynamic parameters following targeted PA ablation in the IPAH patient with positive vasoreactive testing should be confirmed in larger studies.

## Figures and Tables

**Table 1:** Patient clinical characteristics.

	Age, years	PAH classification	PAH therapy	Concomitant diseases	Time from PAH symptoms, years	History of syncope	Loop diuretics
Patient 1	40	IPAH long-term responder to CCB	Amlodipine	Anemia	13	No	No
Patient 2	32	IPAH	Sildenafil + ambrisentan	No	1.3	Yes	Yes
Patient 3	41	IPAH long-term responder to CCB	Diltiazem	Exogenous allergic alveolitis	2	No	No

IPAH: idiopathic pulmonary arterial hypertension; FC (WHO): functional class of pulmonary arterial hypertension according to World Health Organization; 6MWT: 6-min walk distance; CCB: calcium channel blockers.

**Table 2:** Hemodynamic measurements before and after PA ablation and description of PA stimulation reactions.

	Patient 1				Patient 2			Patient 3		
		Baseline	After ablation	12 months	Baseline	After ablation	12 months	Baseline	After ablation	12 months
Hemodynamics	mPAP, mmHg	30	36	18	48	39	48	28	39	40
	PCWP, mmHg	7	9	2	8	3	8	1	3	8
	PVR, dynes *∗* s *∗* cm^−5^	328	344	194	1103	1112	1103	424	291	597
	SVR, dynes *∗* s *∗* cm^−5^	1387	1924	964	1328	659	1711	1639	1607	2040
	CI, L/min *∗* m^2^	2.99	3.2	3.05	1.96	1.72	1.46	2.66	4.19	2.28

PA stimulation reactions	Bradycardia	4			2			3		
	Tachycardia	0			12			3		
	Cough	4			0			2		
	Phrenic nerve capture	3			4			0		

RF ablation	Ablation points, #	13			73			23		
	Total ablation time, s	374.75			928.35			663.02		

mPAP: mean pulmonary artery pressure; PCWP: pulmonary capillary wedge pressure; PVR: pulmonary vascular resistance; SVR: systemic vascular resistance; CI: cardiac index.

**Table 3:** Functional capacity, echocardiography, and laboratory data before and after ablation.

	Patient 1				Patient 2			Patient 3		
		Baseline	6 months	12 months	Baseline	6 months	12 months	Baseline	6 months	12 months
Functional capacity	Functional class (WHO)	II	II	I	III	III	IV	II	II	II
	6MWT, meters	510	546	575	420	300	243	521	510	470
	Peak VO_2_, ml/min/kg	16.9	16.2	15.6	13.8	9.9	11.4	14	13.9	9.7

Echocardiography	sRA, cm^2^	19	18.3	16.3	28	28.5	29	18.3	18.9	20.8
	RV : LV ratio	0.79	0.8	0.7	1.38	1.8	1.65	0.96	0.8	0.85
	RV S′, cm/s	14	14	18	7	7	5	11	12	8

Laboratory	NT-proBNP, pg/ml	118.7	85.4	95	529	1241	1133	56.5	21	94

6MWT: six-minute test walk distance; Peak VO_2_: peak oxygen uptake in cardiorespiratory testing; sRA: right atrium end systolic area; RV : LV: right ventricle to left ventricle diameter ratio; RV S′: tricuspid annular plane systolic velocity; NT-pro BNP: N-Terminal probrain natriuretic peptide.

## Data Availability

The data used to support the findings of this study are included within the article.
